# Keeping pace: the primary cilium as the conducting baton of the islet

**DOI:** 10.1007/s00125-024-06096-6

**Published:** 2024-02-14

**Authors:** Olof Idevall-Hagren, Ceren Incedal Nilsson, Gonzalo Sanchez

**Affiliations:** Department of Medical Cell Biology, Uppsala University, Uppsala, Sweden

**Keywords:** Beta cell, Ca^2+^, cAMP, G protein-coupled receptor, Hedgehog, Primary cilium, Somatostatin, Type 2 diabetes

## Abstract

Primary cilia are rod-like sensory organelles that protrude from the surface of most mammalian cells, including the cells of the islet, and mounting evidence supports important roles of these structures in the regulation of beta cell function and insulin secretion. The sensory abilities of the cilium arise from local receptor activation that is coupled to intrinsic signal transduction, and ciliary signals can propagate into the cell and influence cell function. Here, we review recent advances and studies that provide insights into intra-islet cues that trigger primary cilia signalling; how second messenger signals are generated and propagated within cilia; and how ciliary signalling affects beta cell function. We also discuss the potential involvement of primary cilia and ciliary signalling in the development and progression of type 2 diabetes, identify gaps in our current understanding of islet cell cilia function and provide suggestions on how to further our understanding of this intriguing structure.

**Graphical Abstract:**

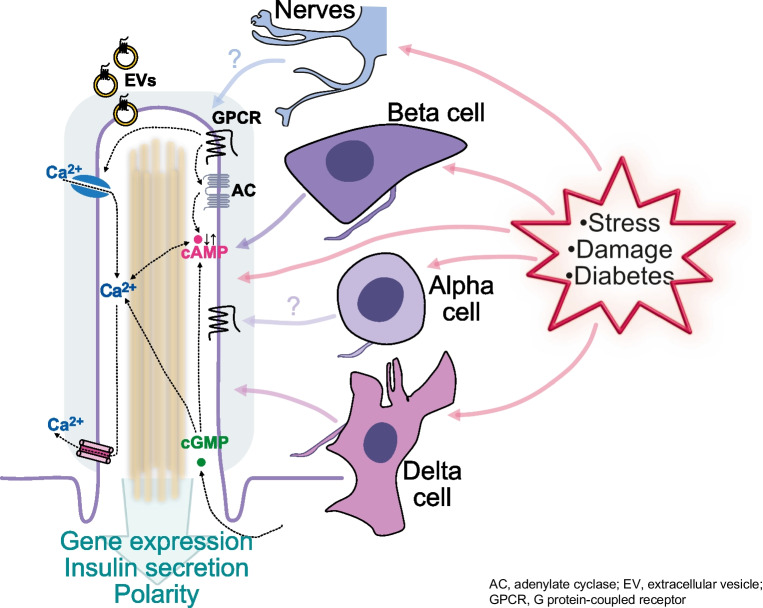

**Supplementary Information:**

The online version contains a slideset of the figures for download available at 10.1007/s00125-024-06096-6.

## Introduction

**Fig. 1:**
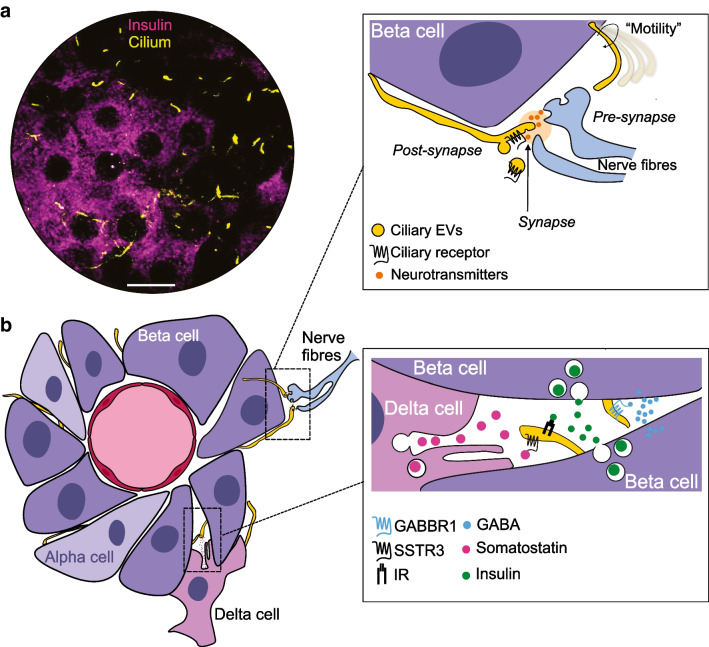
Primary cilia as sensors of the islet environment. (**a**) Confocal microscopy image of a mouse islet immunostained for insulin (magenta) and the primary cilia marker ARL13B (yellow). Scale bar: 10 µm (**b**) Islet beta cells are polarised with a basal domain facing islet blood vessels and lateral domains engaged in cell–cell contacts and where primary cilia exit the cell and project towards the apical domain. The primary cilia are sensory organelles that can detect insulin, somatostatin and gamma-aminobutyric acid (GABA) through cilia-localised receptors. The primary cilia may also be used to detect neurotransmitter released from islet nerve fibres, perhaps in a mode resembling synaptic transmission, where the cilium constitutes the post-synaptic compartment. The cilia can not only detect signals, but also transmit information in the form of extracellular vesicles. This figure is available as part of a downloadable slideset

The islets of Langerhans are pancreatic micro-organs tasked with maintaining whole-body glucose homeostasis. This is accomplished through the release of glucoregulatory hormones, with each of the five different islet cell types releasing a specific hormone that has both systemic and local auto- and paracrine functions. Insulin-secreting beta cells comprise the largest group of islet cells and they establish functional connections with other endocrine cells and islet endothelial cells. The beta cells are polarised, with the basal domain facing islet capillaries and forming a primary site for insulin granule exocytosis, and the lateral domain being involved in cell–cell contact formation [1, 2]. The lateral domain is also the site where a primary cilium exits and projects towards the apical domain, away from the vasculature (Fig. 1). The beta cell primary cilium is a rod-shaped organelle with a fixed diameter of around 200 nm and a variable length in the range of 3–10 µm. Most beta cells contain a single cilium, but occasionally two cilia emerge from a common ciliary pocket [3–5]. The core of the cilium is formed by nine microtubule doublets enclosed by a lipid bilayer that is continuous with the plasma membrane, yet has a unique lipid and protein composition. Its rich abundance of receptors and ion channels provides the cilium with distinct sensing abilities, and it is often referred to as a cellular antenna. The apical positioning of the cilium postulates that it would preferentially sense islet-derived factors, and its receptor repertoire strengthens this assumption [4, 6–8] (Fig. 1b). Beta cell primary cilia also interact with intra-islet axons in an arrangement resembling that between neuronal primary cilia and presynaptic compartments in the central nervous system [5, 9, 10] (Fig. 1b). Defects in primary cilia structure or function can cause ciliopathies, a group of diseases characterised by metabolic abnormalities and obesity. Although only a few of these pathologies are accompanied by insulin secretion defects and diabetes, primary cilia have attracted attention in the diabetes research field since the discoveries that beta cell-selective cilia loss in mice causes impaired insulin secretion and glucose intolerance, indicating bona fide roles of primary cilia in major beta cell pathways [6, 7, 11] (reviewed in [12, 13]).

Diabetes is associated with both intrinsic dysregulation of beta cell function and changes in the local islet microenvironment. The primary cilium, as a sensor of the islet environment, may detect such changes, and dysfunctions in this sensory mechanism may therefore reduce the beta cells’ ability to adapt to these changes. The primary cilium is equipped with many receptors, yet has a limited set of proteins for signal propagation. It may therefore play an important role in signal integration to reduce the complexity of the islet signalling landscape by signal transformation. The primary cilium can detect both acute changes, which could provide feedback to dial up or down hormone secretion, and more sustained changes, which could be used for transcriptional reprogramming and long-term adaptation. Here, we will review recent literature on the role of primary cilia as regulators of beta cell function and provide our view of this fascinating organelle.

## Primary cilia as sensors of the islet microenvironment

### Ciliary receptor signalling

**Fig. 2:**
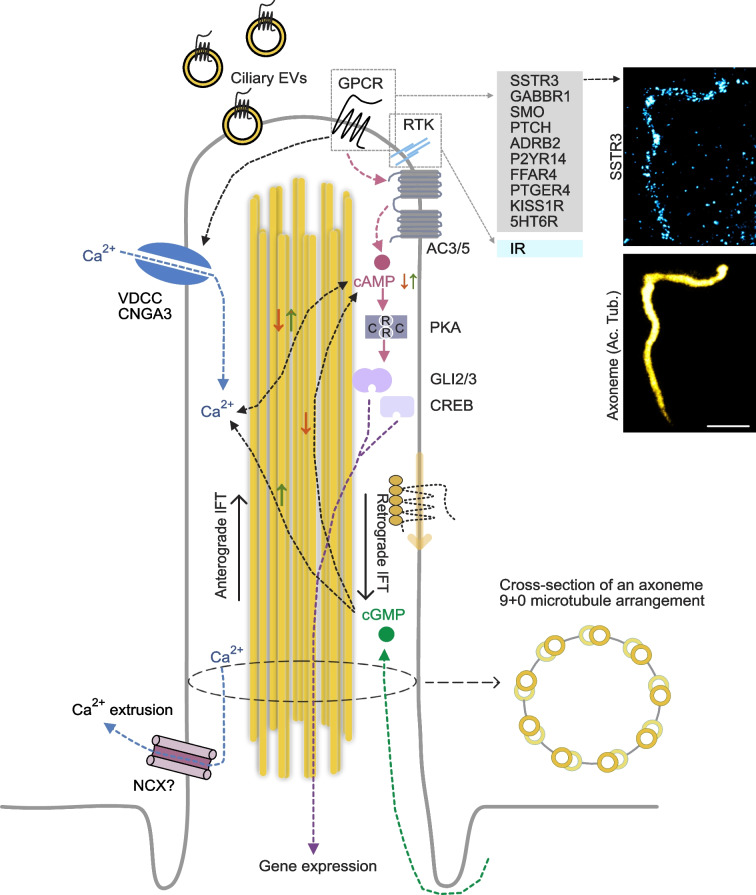
Signal transduction in the beta cell primary cilium. Numerous receptors, primarily G protein-coupled receptors, localise to beta cell primary cilia, and receptor abundance is controlled by anterograde and retrograde intraflagellar transport along ciliary microtubules that are arranged in nine distal doublets (axoneme). The ciliary receptors couple to adenylate cyclase activation or inhibition with resulting cAMP production or degradation, respectively. Ca^2+^ is also an important ciliary second messenger that enters the cilia via cilia-localised ion channels, and the cAMP and Ca^2+^ pathways are intricately connected and also modulated by cGMP, which can enter the cilium from the cytosol. The best-characterised cilia output signal is the family of GLI transcription factors, which are activated by cAMP lowering and exit the cilium to control gene expression, but the activity of other transcription factors can also be modulated in a cilia-dependent manner. The stimulated emission depletion microscopy images show the distribution of SSTR3 (blue) in a primary cilium (yellow) from a mouse islet beta cell. Scale bar: 1 µm. 5HT6R, Serotonin receptor 6; AC, adenylate cyclase; Ac. Tub, acetylated tubulin; ADRB2, beta-2 adrenergic receptor; C, catalytic subunit of PKA; CNGA3, cyclic nucleotide-gated ion channel A3; CREB, cAMP-responsive element-binding protein; EV, extracellular vesicle; GABBR1, GABA_B1_ receptor; GLI, zinc finger protein GLI; IFT, intraflagellar transport; IR, insulin receptor, KISS1R, KISS1-derived peptide receptor; NCX, Na^+^/Ca^2+^ exchanger; P2YR14, P2Y purinoceptor 14; PKA, protein kinase A; PTCH, Patched; PTGER4, prostaglandin EP4 receptor; R, regulatory subunit of PKA; RTK, receptor tyrosine kinase; SMO, Smoothened; VDCC, voltage-dependent Ca^2+^ channel. This figure is available as part of a downloadable slideset

Primary cilia probe the local environment and respond to changes via cilia-localised receptors. These receptors can either be exclusively found in the cilium or be enriched in relation to the plasma membrane (Fig. 2). The receptors gain entry into the ciliary compartment through interactions between cilia-targeting sequences in the receptors and gatekeeper proteins at the cilia base [14]; receptors can also be actively removed from the cilium though a β-arrestin-dependent mechanism or via disposal in cilia-derived extracellular vesicles (EVs) [15, 16]. This arrangement allows cilia to vary receptor composition over time. It is worth noting that even a small number of receptors may be sufficient to initiate intra-ciliary signal transduction. This is, at least in part, due to the cylinder-like shape of the cilium, which gives it a very large surface-to-volume ratio that allows for efficient coupling to downstream signalling events, and the small volume (around 1/10,000th of the cell body), which enables strong signal amplification through the action of a relatively small number of effector molecules. Numerous receptors localise to the primary cilium of beta cells, including somatostatin receptor type-3 (SSTR3) and free fatty acid receptor 4 (GPR120, also known as FFAR4) [7, 8] (Fig. 2). Using beta cells lacking cilia or with a defective cilia receptor trafficking machinery, it has been shown that some, if not all, of the effects of both SSTR3 and FFAR4 on insulin secretion modulation are exerted via cilia-localised receptors [4, 6–8, 17]. Many receptors or receptor families, for example SSTRs, are also present on the plasma membrane, suggesting that some ligands may trigger parallel canonical and ciliary pathways, which may increase the information content carried by a given ligand. For example, in mice, NEFAs amplify insulin secretion in a cAMP-dependent manner through activation of free fatty acid receptor-1 (GPR40, also known as FFAR1) and GPR120, and use of selective agonists shows that the two receptors act via distinct, additive mechanisms [18]. Intriguingly, GPR120 localises to primary cilia and this localisation is required for augmentation of insulin secretion, while GPR40 is plasma membrane localised and operates independently of the cilium [8], suggesting parallel processing of a common signal. However, such parallel processing may not even require distinct receptor isoforms but instead depend on differences in receptor activation and coupling in the ciliary microenvironment [19]. For example, 1 nmol/l insulin was sufficient to induce insulin receptor substrate 1 (IRS1) phosphorylation in the primary cilia of adipocytes, while 100 nmol/l was required for phosphorylation of plasma membrane-localised IRS1 [20]. Similarly, very low concentrations of gamma-aminobutyric acid (GABA; 1 nmol/l) activate ciliary GABA_B_ receptors and induce ciliary Ca^2+^ signalling [4], whereas micromolar concentrations of GABA are required to activate plasma membrane GABA_B_ receptors to suppress insulin secretion [21]. Additional specificity in ciliary signalling may be achieved if the ligand is locally delivered to the cilium, as was recently shown in hippocampal neurons, where vesicular serotonin is delivered towards primary cilia in a synapse-like configuration [9]. In this scenario, the primary cilium functions as a post-synaptic compartment (see Fig. 1b). In fact, the cilium has been proposed to be the evolutionary origin of the neuronal post-synapse, and many post-synaptic-like proteins appeared in early ciliated cells, long before the establishment of a nervous system. In these cells, the cilium functioned as a synapse with the environment, and it is possible that this feature remains in primary cilia of a metazoan [22].

### Cilia motility

A small number of cell types are equipped with motile cilia, which are a special type of primary cilia characterised by a central microtubule doublet and the presence of dynein motor proteins. Central microtubule doublets have been observed in beta cell primary cilia [23, 24], and glucose triggers regular, beating-like movements in beta cell cilia of isolated islets that are important for normal insulin secretion and depend on cilia-specific motor proteins [24]. However, recent 3D ultrastructural examination of beta cell cilia shows that the central microtubule doublets are in fact outer doublets displaced towards the cilia centre, which is an arrangement that is incompatible with microtubule-based cilia motility [5]. Primary cilia motility can also be induced by ATP-dependent actomyosin force generation in the cytosol that couples to primary cilia via the basal body [25]. Beta cell primary cilia have an actin-rich base [3] and glucose metabolism may generate the ATP required for myosin-based contraction, but this requires experimental validation. It also remains to be shown to what extent such movements can occur in situ, where primary cilia appear to have exceptionally little freedom to move [5]. However, it is intriguing to think that motility may be yet another mechanism whereby cilia enhance their ability to probe and sense the local islet environment.

### Cilia-derived extracellular vesicles

In addition to receiving signals, primary cilia are also a source of bioactive EVs, which are generated by actin-dependent ectocytosis or by exocytosis of multivesicular bodies. EVs carry distinct signalling factors, including receptors and miRNA, and participate in intercellular communication [16, 26]. The composition of ciliary EVs is different from the composition of EVs released from the cell body, indicating that they represent a distinct functional pool [27], and the composition of ciliary EVs is altered in ciliopathies, suggesting that such EVs may be relevant biomarkers in disease [28]. The fact that cilia can release EVs expands their functional repertoire, providing the possibility of operating not just as signal receivers but also as signal emitters. The release of ciliary EVs appears to be a general feature of primary cilia, but to the best of our knowledge this has not yet been observed within islets.

## Coupling ciliary receptors to downstream signalling pathways

### Ca^2+^ signalling in primary cilia

**Fig. 3:**
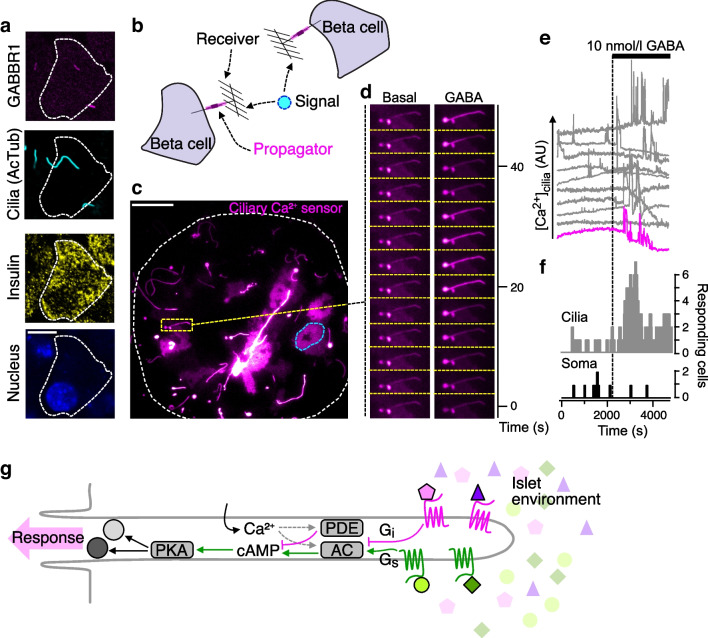
GABA signalling in the beta cell primary cilium. (**a**) Confocal microscopy images of a mouse islet beta cell immunostained for GABBR1 (magenta), acetylated tubulin (AcTub; cilium; cyan), insulin (yellow) and DAPI (nucleus; blue). GABA receptors have a distinct localisation in a ciliary sub-compartment. Scale bar: 5 µm (**b**) The primary cilium functions as a signal receiver that propagates the signal in the form of second messengers, e.g. Ca^2+^. (**c**) TIRF microscopy image of the footprint of a mouse islet expressing the cilia-enriched Ca^2+^ sensor 5HT6-G-GECO1 (a cilium is encircled in yellow and a cell body is outlined in blue). Scale bar: 10 µm. (**d**) Time-series images of Ca^2+^ concentration changes in a cilium under basal conditions and after addition of 10 nmol/l GABA. Note that GABA induces ciliary Ca^2+^ signalling. (**e**) Ca^2+^ concentration changes in 10 cilia from one mouse islet shows that the addition of 10 nmol/l GABA triggers Ca^2+^ signalling in most cilia. Trace in magenta is from the cilium in (**d**). (**f**) Cumulative Ca^2+^ responses in all cilia (top; grey) and cell bodies (bottom; black) from a mouse islet in response to 10 nmol/l GABA. GABA exclusively triggers Ca^2+^ signalling in the cilia. (**g**) Proposed model for cilia signalling in the islet. The cilium is equipped with multiple receptors that can detect specific ligands within the islet interstitium. Simultaneous activation of multiple receptors converges on cAMP formation and PKA-dependent activation of downstream effector proteins, and the response can be modulated by intra-ciliary Ca^2+^ signalling. AC, adenylate cyclase; AU, arbitrary unit; GABBR1, GABA_B1_ receptor; PDE, phosphodiesterase; PKA, protein kinase A. This figure is available as part of a downloadable slideset

Activation of ciliary receptors initiates local signal transduction. Recent developments in genetically encoded biosensors have enabled probing of signal transduction in primary cilia and have led to the conclusion that both Ca^2+^ and cAMP are bona fide ciliary second messengers [29, 30]. Ca^2+^ can enter the primary cilium through numerous channels in non-excitable cells, including members of the transient receptor potential (TRP) and polycystin families [31], and it has been shown that constitutive Ca^2+^ influx maintains high basal ciliary Ca^2+^ concentration [29]. This situation appears to be different in the excitable beta cell, where the resting Ca^2+^ concentration is comparable between cilium and cytosol, and where Ca^2+^ diffusion to and from the cytosol is actively prevented through efficient extrusion at the ciliary base [4]. This enables the cilium to generate rapid, intrinsic Ca^2+^ signals. GABA, which is released non-vesicularly from beta cells, binds to GABA_B1_ receptors on the cilia surface and initiates local ciliary Ca^2+^ influx through a G_i_-independent mechanism that involves activation of voltage-dependent Ca^2+^ channels [4] (Fig. 3). The Ca^2+^ signals are characterised by rapid on- and off-kinetics and are often restricted to ciliary sub-compartments. In other cell types, ciliary Ca^2+^ has been shown to negatively regulate cilia length, which may attenuate ciliary sensing abilities, and to directly modulate ciliary receptor signalling [32, 33], yet direct evidence that Ca^2+^ acts as a rapid second messenger, similar to its function in the cytosol, is lacking. Some clues may come from studies of Ca^2+^ signalling in dendritic spines, which are post-synaptic structures that share many properties with primary cilia, including a high surface-to-volume ratio, small size, isolation from the cell body and chemosensory abilities [34]. Ca^2+^ directly contributes to spine depolarisation, but also activates a wide variety of Ca^2+^-sensitive proteins, for example Ca^2+^/calmodulin-dependent protein kinase II (CaMKII) and calcineurin, that regulate synaptic function. Like dendritic spines, primary cilia also constitute a post-synaptic compartment [9, 10] and are rich in Ca^2+^-binding proteins [35], so it seems likely that the two compartments will share some functions.

### Cyclic nucleotide signalling in primary cilia

The short-timescale Ca^2+^ signals in dendritic spines are connected to more sustained events, such as structural plasticity, mediated by cAMP/protein kinase A (PKA). The primary cilium is also a unique cAMP compartment that contains both adenylate cyclases (ACs) and phosphodiesterases (PDEs) as well as PKA, and several of these are directly controlled by Ca^2+^/calmodulin, which might enable local crosstalk between the Ca^2+^ and cAMP pathways [31]. Beta cell primary cilia contain both AC3 and AC5/6, and activation of GPR120 can stimulate ciliary cAMP formation [4, 8, 17]. Given the presence of both G_s_- and G_i_-coupled receptors on the beta cell cilia (Fig. 2), it is likely that both increases and decreases in cilia cAMP occur, and that these in turn control the activity of ciliary PKA. Work in non-excitable cells has shown that cAMP positively controls cilia length through a PKA-dependent mechanism [33] and that decreases or increases in ciliary PKA activity can lead to activation of GLI family zinc finger (GLI)-dependent transcription and cAMP-responsive element-binding protein (CREB)-dependent transcription, respectively. The regulation is receptor-independent and directly governed by ciliary cAMP [36, 37], indicating that primary cilia function as signal integrators, in which complex signals in the local environment are detected by ciliary G protein-coupled receptors (GPCRs) and transformed to a simple, perhaps even binary, signal in the form of cAMP/PKA. This signal can then be further tuned by simultaneous changes in the ciliary Ca^2+^ concentration (Fig. 3g). Another second messenger of potential importance in primary cilia is cGMP. This nucleotide is a prominent messenger in photoreceptor sensory cilia in the retina, which are a special type of cilia that both structurally and functionally resemble primary cilia. cGMP is generated by soluble or transmembrane guanyl cyclases and is a known regulator of insulin secretion, acting either via protein kinase G (PKG) or through modulation of phosphodiesterases with ensuing cAMP concentration changes [38]. Beta cell primary cilia, similar to photoreceptor sensory cilia, contain cyclic nucleotide-gated ion channels that promote Ca^2+^ influx on binding of cGMP but not cAMP [4]. cGMP enters the cilium via diffusion from the cytosol, and evidence of local cGMP production in the primary cilium is lacking. This may thus be a mechanism whereby the cytosol can gain control over ciliary signalling, and cGMP may be particularly suitable as it has the ability to influence both ciliary Ca^2+^ and ciliary cAMP. The recent observation that neuronal cell polarity and migration are regulated by the ciliary cAMP/cGMP ratio is another indication that cGMP is a bona fide ciliary second messenger [39]. Advances in the development of cilia-targeted sensors for Ca^2+^ and cyclic nucleotides together with opto- or chemogenetic control of second messenger generation should make it possible to determine the intricate interplay between these key ciliary messenger molecules.

## Regulation of islet cell function through primary cilia signalling

### Lessons from mouse models

The most straightforward way to dissect the role of primary cilia in the regulation of beta cell function is to generate beta cells that lack this organelle. Current models rely on beta cell-specific deletion of the *Ift88* gene using *Pdx1*-CreER (referred to as βICKO mice) or *Ins1*-CreERT2 (referred to as βCKO mice) [7, 11]. While *Ins1* expression is restricted to beta cells, *Pdx1* is also expressed in hypothalamic nuclei [40]. This may complicate interpretations of findings in these mice, especially as neurons in these regions control whole-body energy expenditure and rely on primary cilia for normal function [41]. Loss of beta cell primary cilia postnatally results in the progressive disappearance of glucose-stimulated insulin secretion with accompanying glucose intolerance and a type 2 diabetes-like phenotype. Initially, loss of secretion occurs without accompanying loss of beta cell mass, indicating intrinsic defects, but over time there is a substantial decrease in the number of beta cells [7, 11]. In the βICKO mouse model, the secretory defect is at least partly caused by changes in beta cell polarity that result in an endocytic defect that impairs recycling of ephrin type-A receptor 3 (EphA3) and causes aberrant juxtacrine signalling. Although cell polarity was not investigated in βCKO islets, these appear to have normal juxtacrine regulation of insulin secretion and instead the beta cells exhibit an impaired glucose-induced Ca^2+^ response with loss of regular Ca^2+^ oscillations. The intrinsic changes in βCKO islets were accompanied by an increase in the number of delta cells and hypersecretion of both somatostatin and glucagon at low glucose levels [7], indicating roles of beta cell primary cilia in paracrine islet signalling. While glucagon retained its ability to enhance insulin secretion from βCKO islets, somatostatin lost the ability to suppress secretion, indicating that beta cell cilia are direct targets of somatostatin action. Consistently, short hairpin RNA (shRNA)-mediated loss of SSTR3, the major ciliary somatostatin receptor, renders somatostatin incapable of modulating cytosolic Ca^2+^ signalling and inhibiting insulin secretion [17]. It has also been shown that insulin receptors dynamically localise to beta cell cilia, and that trafficking to the cilium is important for downstream Rac-alpha serine/threonine-protein kinase (Akt) phosphorylation [6]. Whether altered insulin receptor signalling contributes to the defective insulin secretion is not known, but the fact that beta cell-specific loss of the insulin receptor phenocopies several defects observed in the βICKO/βCKO mice is consistent with this notion [42].

### Signal propagation from cilium to cell body

The loss-of-function studies discussed in the previous section clearly show that normal beta cell function requires an intact cilium, but how can this tiny protrusion have such profound effects on beta cell function? Given the small volume of the cilium, it is unlikely that second messenger diffusion into the cytosol will have a major impact beyond the cilia base or its immediate vicinity. Likewise, membrane potential changes are unlikely to propagate from the cilia membrane to the plasma membrane, as these membranes are, at least partially, electrically insulated from each other [43]. Instead, the cilium may be involved in more long-term control of beta cell function. Hedgehog (Hh) is a morphogen secreted by beta cells that binds to its ciliary receptor, Patched, resulting in receptor exit and the reciprocal entry of the GPCR Smoothened, which in turn lowers ciliary cAMP and suppresses ciliary PKA activity. Ciliary PKA typically maintains GLI2/3 transcription factors in an inactive state, and inhibition of PKA causes processing of GLI2/3 to active forms that exit the cilium and enter the nucleus to regulate transcription of target genes. Deletion of beta cell cilia, combined with GLI2 overexpression, causes exacerbated Hh signalling that leads to reduced insulin secretion and glucose intolerance, at least in part due to reduced expression of beta cell identity markers (*Pdx1*, *Nkx6-1*, *Mafa*, *Neurod1* [also known as *Neurod*], *Neurog3* [also known as *Ngn3*]) and upregulation of the precursor cell markers *Hes1* and *Sox9* [44]. Similarly, it has been shown that Hh signalling in alpha cells is required to maintain cell identity, and that loss of Smoothened induces the expression of beta cell genes and the production of insulin [45]. Recent studies also indicate that the classical hedgehog response can be tuned by the activation of other ciliary receptors, with G_i_-coupled receptor activation enhancing the response and G_s_-coupled receptor activation antagonising it [46]. It therefore seems likely that islet-derived factors, for example somatostatin and GABA [4, 17], will crosstalk with the Hh pathway to maintain islet cell identity and functionality, and that disturbances in this regulation, for example changes in cilia structure or composition, will lead to loss of this paracrine control.

## Changes in primary cilia function in type 2 diabetes: cause or consequence?

Ciliopathies are rare monogenic disorders caused by loss-of-function mutations in cilia genes that either result in complete loss of primary cilia or render the cilia dysfunctional. Most ciliopathies are associated with obesity, but with a few exceptions (Bardet–Bieldl syndrome and Alström syndrome) rarely lead to development of diabetes. However, common variants in ciliopathy genes, which do not cause penetrant disease, show a strong correlation with complex disease pathology [47]. For example, hypomorphisms in the cilia gene *Rpgrip1l*, found within the fat mass and obesity-associated FTO locus, have been shown to cause obesity [48]. These observations indicate a plausible mechanism for how polymorphisms influencing the expression of one or more cilia genes might contribute or predispose to multifactorial diseases such as diabetes. The putative connection between cilia function and insulin secretion has led to the establishment of mouse and beta cell models lacking primary cilia, and these all present with impaired insulin secretion as discussed above [6, 7, 11]. The similarities between loss-of-cilia phenotypes and beta cell dysfunction in type 2 diabetes could be an indication of a role for cilia in disease development or progression. There are reports of beta cell cilia loss in rodent models of type 2 diabetes [6, 49], but there is no evidence that the same occurs in human type 2 diabetes. Observations that the expression of cilia genes involved in proliferation, cell cycle control and cilia motility is reduced in type 2 diabetes [24, 49] could be an indication that cilia structure and function are altered, but does not provide causality. In another study on early type 2 diabetes it was found that cilium assembly, organisation and motility were among the most enriched pathways based on differential gene expression, but in this case the changes involved upregulation of cilia gene expression, which also correlated with a higher abundance of ciliated islet cells. These changes were associated with a mild defect in insulin secretion but normal insulin production [50], somehow resembling the early phenotype in the βICKO mouse model [11]. It is possible that these disparate results represent distinct functions of primary cilia in type 2 diabetes, involving initial upregulation of cilia genes and later loss-of-cilia function that coincides with disease progression. A cilia gain-of-function mouse model would therefore be an important complement to the established loss-of-function models for assessing the contribution of primary cilia to type 2 diabetes. It will also be important to understand what drives changes in cilia gene expression. Key candidates belong to the regulatory factor binding to the X-box (RFX) family of transcription factors, which are involved in both cilia formation and endocrine cell specification during development [51, 52]. The RFX proteins, like the Hh pathway, also have important functions in adult beta cells. RFX6, for example, controls glucose homeostasis, the insulin secretory pathway and repression of disallowed genes [53]. *RFX6* is also mutated in autosomal recessive syndrome of neonatal diabetes [52] and shows reduced expression in beta cells in early type 2 diabetes [50]. Knockdown of *RFX6* in clonal beta cells also leads to dysregulation of cilia genes, which resembles the transcriptional changes observed in early type 2 diabetes [50], and dysregulation of *Rfx* genes and ciliogenesis has been observed in metabolically stressed cells [54]. There is also indirect evidence of a link between cilia and type 2 diabetes. Risk alleles in the *ADCY5* locus result in reduced expression of AC5 and reduced beta cell connectivity and impaired insulin secretion [55, 56]. Moreover, recent human genetics studies have shown that mutations in *ADCY3* are associated with type 2 diabetes [57]. Both AC5 and AC3 localise to the cilia, and the type 2 diabetes-associated changes are likely to influence the cilia’s ability to generate cAMP. Intriguingly, reduced beta cell connectivity is also a feature of type 2 diabetes islets that stems from loss of leader beta cells, which is a cellular subtype characterised by metabolic and transcriptional immaturity, close proximity to islet delta cells and short cilia [58, 59]. As primary cilia cAMP signalling is central to the Hh pathway, a key regulator of beta cell maturity, it is possible that altered ciliary input, detection or signal propagation may contribute to loss of islet connectivity in type 2 diabetes. The connection between leader cells, islet connectivity and cilia signalling warrants further investigation.

Primary cilia are information transducers that sense the environment and provide feedback to the beta cell through transcriptional regulation to enable adaptive responses. As such, cilia may play a crucial role in type 2 diabetes development as a failing link between intrinsic regulation and changes within the islet microenvironment. Strategies aimed at restoring or interfering with cilia signalling therefore represent a previously unexplored therapeutic approach to prevent beta cell failure or restore beta cell function in type 2 diabetes.

## Concluding remarks and outlook

Primary cilia are now considered important regulators of cell function and the list of cell types and functions in which primary cilia signalling is involved is growing rapidly and now includes pancreatic beta cells and regulation of insulin secretion. Although the requirement for a structurally intact cilium for normal beta cell function is well-established, it is far from clear what the cilium senses, in what form signals are transmitted to the rest of the cell and to what extent ciliary signalling is altered in any form and stage of diabetes. It will also be important to determine the role of primary cilia in the other endocrine cell types of the islets and to understand how ciliary signalling, which occurs in parallel with canonical signalling, contributes to auto- and paracrine regulation of islet function. Both alpha and delta cells express structural and functional cilia genes, including *Arl13b*, *Kif3a* and *Ift88* [60], and ultrastructural examination has shown the presence of primary cilia on both cell types [5, 61, 62]. In the case of alpha cells, their cilia can physically interact with both adjacent beta cells and intra-islet axons, indicating a sensory function [5], yet with one exception [45] there are no functional studies on primary cilia in either alpha cells or delta cells. As cilia are environmental sensors, experiments in which the islet environment is kept as intact as possible, such as when using acute pancreatic slices, will be an important complement to studies on isolated islets. The recent developments in molecular tools for real-time detection and manipulation of ciliary signalling, methods for high-resolution imaging of cilia and advanced mouse models should provide the research community with the necessary tools to elucidate the roles of primary cilia in health and in diabetes. And in the meantime, the cilia conduct and the islet cells follow.

### Supplementary Information

Below is the link to the electronic supplementary material.

Slideset of figures (PPTX 2.17 MB)

## Abbreviations

AC: Adenylate cyclase
EV: Extracellular vesicle
FFAR1: Free fatty acid receptor 1
FFAR4: Free fatty acid receptor 4
GABA: Gamma-aminobutyric acid
GLI: GLI family zinc finger
GPCR: G protein-coupled receptor
Hh: Hedgehog
PKA: Protein kinase A
RFX: Regulatory factor binding to the X-box
SSTR3: Somatostatin receptor type-3
