# Supplementary material for: Keeping pace: the primary cilium as the conducting baton of the islet
Source: Diabetologia. 2024 Feb 14;67(5):773–82. doi: 10.1007/s00125-024-06096-6 (PMC10955035; doi:10.1007/s00125-024-06096-6)
Supplement: Supplementary file 1 — Slideset of figures (PPTX 2.17 MB) [file 125_2024_6096_MOESM1_ESM.pptx]

## Slide 1
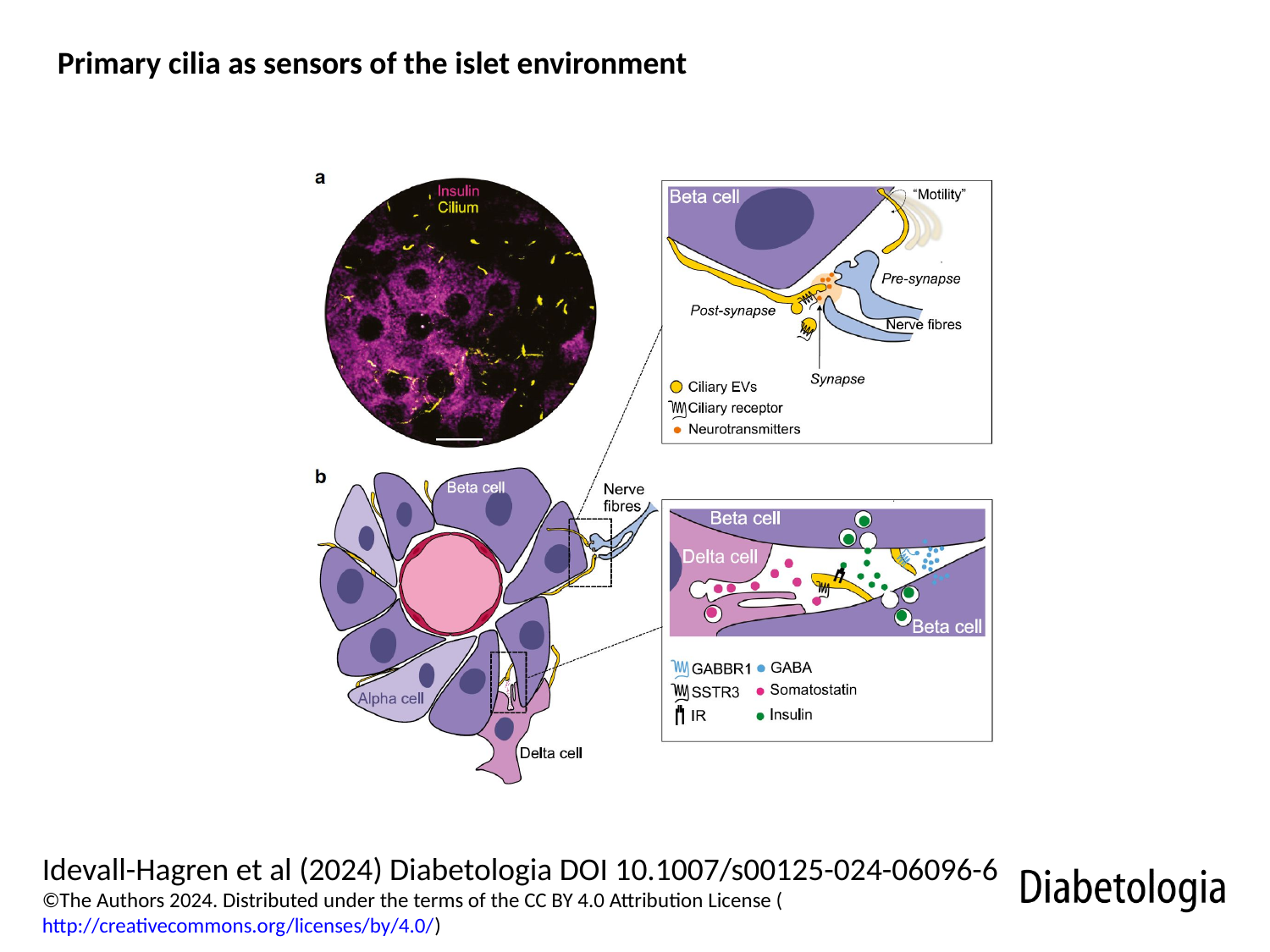

Primary cilia as sensors of the islet environment
Idevall-Hagren et al (2024) Diabetologia DOI 10.1007/s00125-024-06096-6
©The Authors 2024. Distributed under the terms of the CC BY 4.0 Attribution License (http://creativecommons.org/licenses/by/4.0/)

## Slide 2
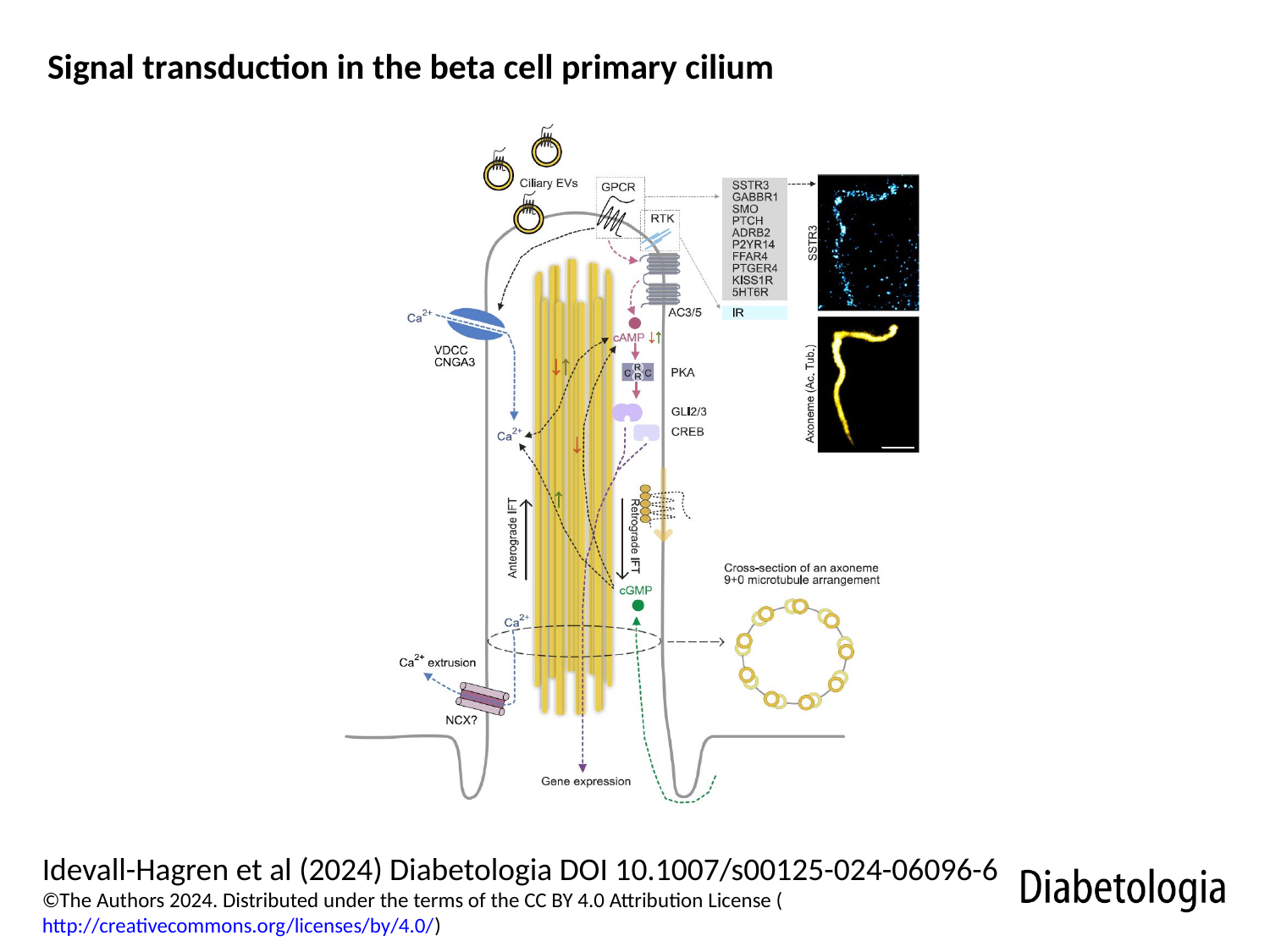

Signal transduction in the beta cell primary cilium
Idevall-Hagren et al (2024) Diabetologia DOI 10.1007/s00125-024-06096-6
©The Authors 2024. Distributed under the terms of the CC BY 4.0 Attribution License (http://creativecommons.org/licenses/by/4.0/)

## Slide 3
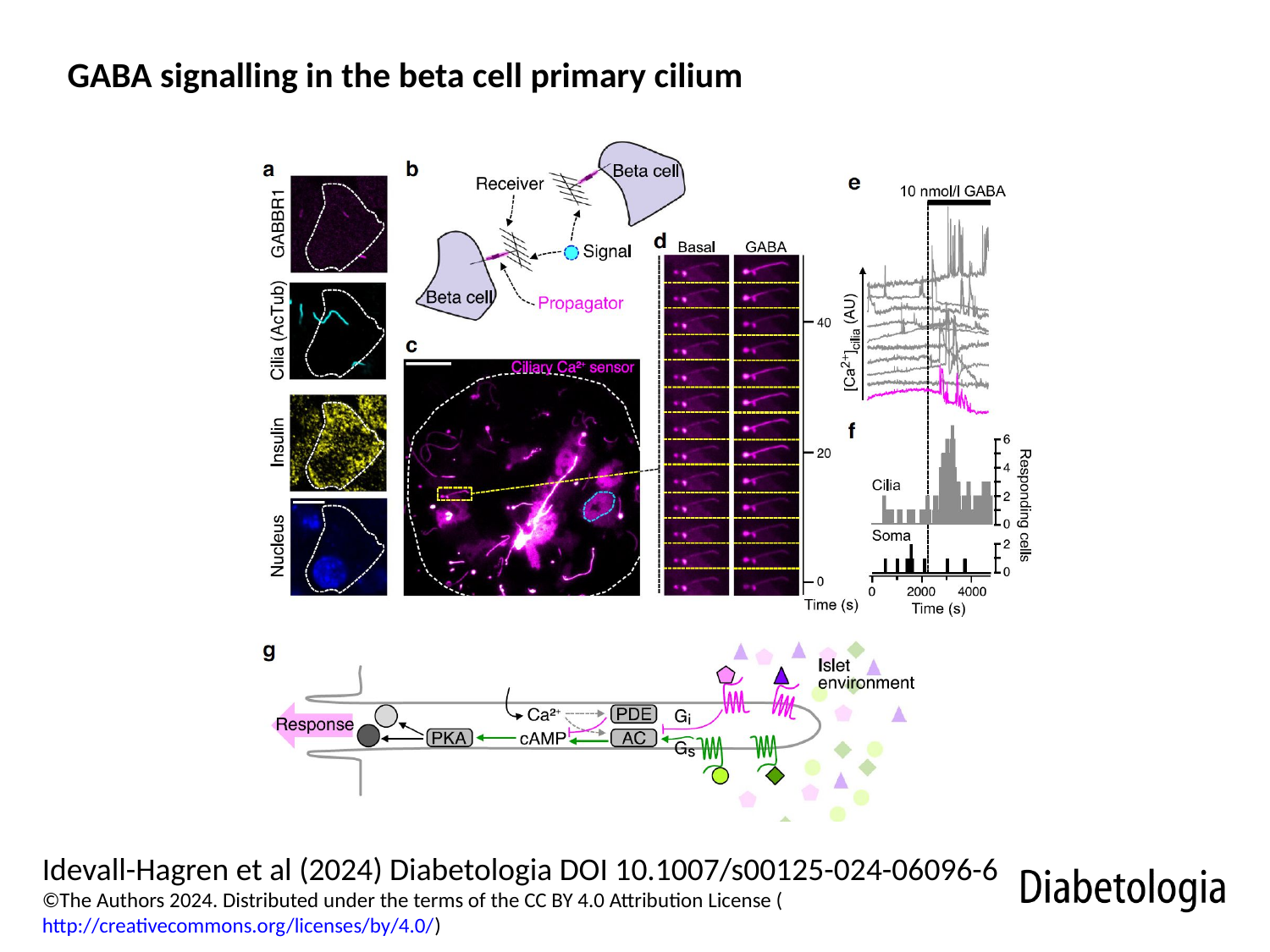

GABA signalling in the beta cell primary cilium
Idevall-Hagren et al (2024) Diabetologia DOI 10.1007/s00125-024-06096-6
©The Authors 2024. Distributed under the terms of the CC BY 4.0 Attribution License (http://creativecommons.org/licenses/by/4.0/)
